# A rare electrocardiographic sign of acute inferior myocardial infarction

**DOI:** 10.1007/s12471-023-01815-x

**Published:** 2023-09-12

**Authors:** Mirte Hoevenaars, Robert J. van Geuns, Niels van Royen, Peter Damman

**Affiliations:** Department of Cardiology, Radboud University Medical Centre, Nijmegen, The Netherlands

**Fig. 1:**
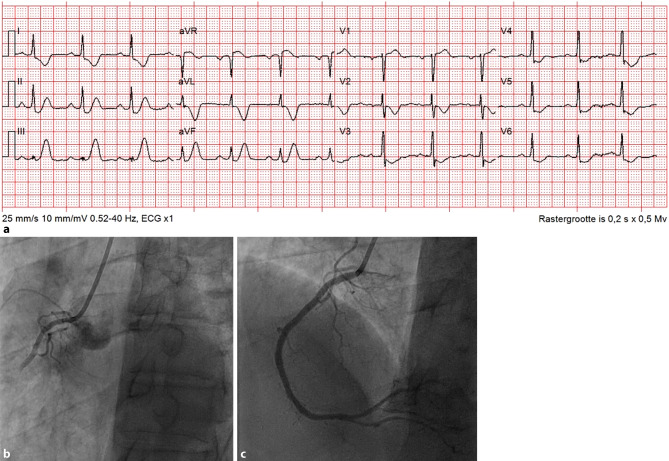
**a** Electrocardiogram at first medical contact. **b** Angiography showing proximal occlusion of the right coronary artery. **c** Angiography after primary percutaneous intervention

A 62-year-old man attended our emergency department with acute chest pain. The electrocardiogram (ECG) showed 2 mm ST-segment depression at the J point with tall, peaked T‑waves in inferior leads, 2 mm ST elevation in aVR and reciprocal depressions in leads I and aVL (Fig. 1a). These features resemble ‘de Winter pattern’ ECG abnormalities: upsloping ST-segment depression at the J point in leads V1–V6 with tall, positive and symmetrical T waves. This is recognised as a sign of proximal left anterior descending coronary artery occlusion, observed in a minority of patients with acute anterior myocardial infarction [1]. This ECG pattern has rarely been described in inferior ST-segment elevation myocardial infarction [2].

We performed urgent coronary angiography, showing a proximal occlusion of the right coronary artery (Fig. 1b). After primary percutaneous coronary intervention (Fig. 1c), complaints disappeared and the ECG normalised.

We emphasise the importance of early recognition in order to prevent a delay in treatment.
